# Causalities of war: The connection between type VI secretion system and microbiota

**DOI:** 10.1111/cmi.13153

**Published:** 2020-01-12

**Authors:** Luke P. Allsopp, Patricia Bernal, Laura M. Nolan, Alain Filloux

**Affiliations:** ^1^ National Heart and Lung Institute Imperial College London London UK; ^2^ Department of Biology, Faculty of Sciences Universidad Autónoma de Madrid Madrid Spain; ^3^ Department of Life Sciences, MRC Centre for Molecular Bacteriology and Infection Imperial College London London UK

**Keywords:** bacterial secretion, effectors, Gram‐negative, microbiota, pathogenesis, protein secretion, T6SS, toxins

## Abstract

Microbiota niches have space and/or nutrient restrictions, which has led to the coevolution of cooperation, specialisation, and competition within the population. Different animal and environmental niches contain defined resident microbiota that tend to be stable over time and offer protection against undesired intruders. Yet fluxes can occur, which alter the composition of a bacterial population. In humans, the microbiota are now considered a key contributor to maintenance of health and homeostasis, and its alteration leads to dysbiosis. The bacterial type VI secretion system (T6SS) transports proteins into the environment, directly into host cells or can function as an antibacterial weapon by killing surrounding competitors. Upon contact with neighbouring cells, the T6SS fires, delivering a payload of effector proteins. In the absence of an immunity protein, this results in growth inhibition or death of prey leading to a competitive advantage for the attacker. It is becoming apparent that the T6SS has a role in modulating and shaping the microbiota at multiple levels, which is the focus of this review. Discussed here is the T6SS, its role in competition, key examples of its effect upon the microbiota, and future avenues of research.

## 1 INTRODUCTION

**Figure 1:**
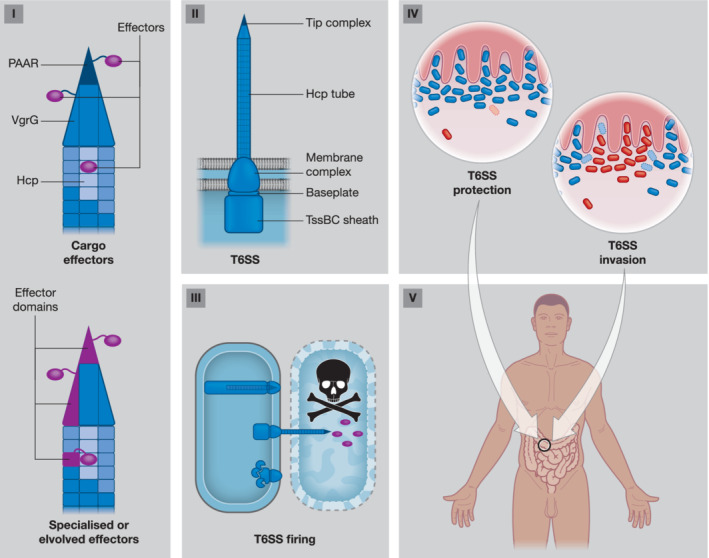
Schematic of the T6SS from host to effector delivery: (I) effectors coupling for T6SS delivery. “Cargo” effectors noncovalently interact with structural components (VgrG/PAAR/Hcp), whereas “specialised” or “evolved” effectors contain additional effector domains within one protein (VgrG/PAAR/Hcp). (II) Protein subcomplexes of the T6SS. (III) Model of contraction‐based firing. The T6SS assembles in an extended conformation, fires, and is then disassembled for recycling. Delivery of effectors (purple) into competing cells results in growth stasis or death. (IV) Antibacterial activity of the T6SS in the intestine. Top panel shows T6SS‐mediated defence by the microbiota (blue) against pathogen (red). Bottom panel shows invasion and killing of microbiota by pathogen. Dotted outlines and pale colour indicate dead cells. (V) Model of human with outline of the intestine focusing on region of interest

**Figure 2:**
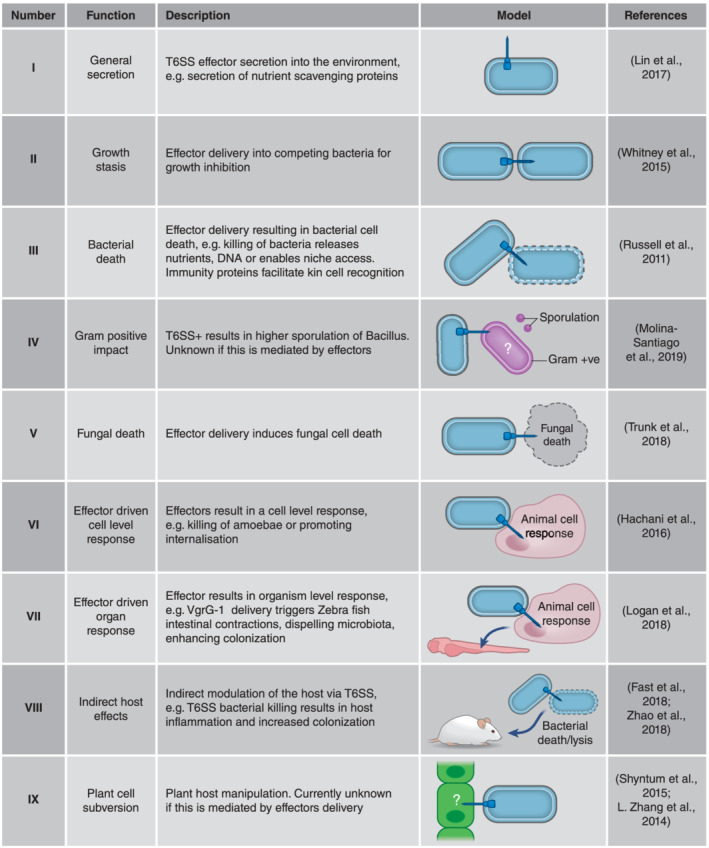
Key functional impact and roles of the T6SS. See text for additional details

The microbiota are a community of microorganisms composed of bacteria, archaea, protists, fungi, and viruses. Within these mixed communities, organisms compete for limited resources and space. These drivers have forced the coevolution of mechanisms of specialisation collaboration and competition. Bacteria are no exception; these social organisms contend for survival and resources for reproduction in the microbiota arena. They have multiple strategies to fight competitors and predators as well as for subverting host cells. In their armament are molecules (e.g., antibiotics, colicins, and siderophores) as well as specialised secretion systems for the export of functional proteins termed effectors. The type VI secretion system (T6SS) delivers effectors into cells using a puncturing mechanism akin to that of bacteriophages (Figure 1; Nguyen et al., 2018). This system is present in more than 25% of all Gram‐negative bacteria including pathogens and environmental symbiotic or commensal microorganisms (Bingle, Bailey, & Pallen, 2008). The T6SS forms three discrete multiprotein subunit structures (Figure 1; Nguyen et al., 2018). The membrane complex TssJLM defines the site of T6SS assembly and enables the baseplate to couple, which facilitates formation of a tail‐like structure. A contractile sheath is composed of repeating subunits of TssB/C (VipA/B) stored in a high‐energy state and wraps the inner tube, composed of Hcp rings. Firing of the T6SS leads to a coordinated collapse of the sheath, which propels the Hcp tube through the membrane complex. At the tip of the Hcp tube is the puncturing device made of a trimer of VgrG proteins and PAAR protein cap, which facilitates puncture of both producing and target cell membranes. Loaded on the secretion machine are two classes of effectors (Figure 1). The “evolved” or “specialised” class has effector domains fused to structural T6SS components, that is, Hcp, VgrG, or PAAR proteins (Coulthurst, 2019; Hachani, Wood, & Filloux, 2016). The “cargo” effectors interact with these aforementioned proteins either directly or through specific adaptors proteins (Coulthurst, 2019). These effectors are delivered extracellularly, into eukaryotic target cells such as amoeba, animal cells, or fungal competitors, or more commonly into bacterial competitors to kill or suppress growth (Figures 1 and 2; Coulthurst, 2019; Hachani et al., 2016; Lin et al., 2017; Trunk et al., 2018).

## 2 T6SS AND ANTIMICROBIAL ACTIVITY

T6SS antibacterial effectors target vital bacterial cellular functions and result in growth stasis or death of competing bacteria (Figure 2). These effectors are encoded in effector–immunity pairs (also called toxin/immunity or effector modules) with the immunity/antidote preventing intoxication from T6SS active siblings (Coulthurst, 2019). The effectors secreted by the T6SSs are classified according to their targets and functions (Russell, Peterson, & Mougous, 2014). Amongst antibacterial effectors targets are nucleic acids cleaved by nucleases (Ma, Hachani, Lin, Filloux, & Lai, 2014; Pissaridou et al., 2018), cell wall degraded by peptidoglycan amidases or hydrolases (Russell et al., 2012), membranes permeabilised by lipases/phospholipases (Russell et al., 2013; Whitney et al., 2013), or pore‐forming toxins (LaCourse et al., 2018; Miyata, Unterweger, Rudko, & Pukatzki, 2013). Other targeted processes are bacterial cell division (e.g., blocking FtsZ polymerisation; Ting et al., 2018; Wood et al., 2019), protein synthesis (Nolan et al., 2019), or availability of dinucleotides such as NADP+ and ATP to cripple prey cells (Ahmad et al., 2019; Whitney et al., 2015). Some T6SS effectors function as metal ion scavenging proteins, which may result in starvation instead of cell damage and impact the community more broadly (Figure 2; Lin et al., 2017).

The delivered effector repertoire determines the T6SS efficacy. The *Pseudomonas aeruginosa* H1‐T6SS has nine experimentally tested effectors that target nucleic acids, peptidoglycan, the inner membrane, protein synthesis, NADP+, and the ATP pools (Ahmad et al., 2019; Hachani, Allsopp, Oduko, & Filloux, 2014; Hood et al., 2010; LaCourse et al., 2018; Nolan et al., 2019; Pissaridou et al., 2018; Russell et al., 2011; Whitney et al., 2014; Whitney et al., 2015). The requirement for many distinct T6SS effectors is debatable. An arsenal of effectors with synergistic biochemical activities may be more potent than higher levels of one specific effector. Producer cells also increase the odds of killing different competitors and overcoming resistance by increasing effectors diversity and their targets. Additionally, certain effectors may have conditional efficacy and be more impactful under certain environmental conditions (e.g., low pH; LaCourse et al., 2018). Finally, trans‐kingdom effectors target both eukaryotic and prokaryotic cells, enabling greater functionality for lower production cost (Jiang, Waterfield, Yang, Yang, & Jin, 2014). Bioinformatic approaches focusing on conserved motifs, proximity to known T6SS components, or global genomic and proteomic approaches continue to uncover additional effectors, indicating that we are underestimating their number and thus their functions/cellular targets (Coulthurst, 2019; Jana, Fridman, Bosis, & Salomon, 2019; Nolan et al., 2019). T6SS‐harbouring microorganisms also have multiple independent T6SS clusters; for example, *P. aeruginosa* encodes three and *Burkholderia* up to six (Schell et al., 2007). Overall, this suggests that we are likely scratching the surface of what these systems are capable of.

With this broad range of functions, it stands to reason that the T6SS will be employed by bacteria to help gain control of their environment/host or to facilitate community balance by eliminating cheaters and foes (Figure 2).

## 3 THE T6SS MODULATES THE EVOLUTION OF POLYMICROBIAL COMMUNITIES

A community of bacteria or even multiple strains of one bacterium can be shaped by the T6SS on the basis of the effector modules present. As opposed to the core T6SS structural components, which are typically highly conserved, sequence divergence is frequently observed for effector modules even within species (Chatzidaki‐Livanis, Geva‐Zatorsky, & Comstock, 2016; Pissaridou et al., 2018; Unterweger et al., 2014). Antibacterial effector–immunity proteins enable social interactions through recognition of self when the same sets of effector–immunity proteins are expressed in different strains but can drive antagonism if different (Figure 2). Opposing strain swarms of *Proteus mirabilis* form T6SS‐dependent “battle lines” of segregation called Dienes lines (Alteri et al., 2017). In *Vibrio cholerae*, related families of effectors (>30% identity) are associated with immunity proteins whose variation is far greater (Kirchberger, Unterweger, Provenzano, Pukatzki, & Boucher, 2017; Unterweger et al., 2014). Combinations of distinct effector modules within a strain further contribute to interstrain incompatibility and confer a significant competitive advantage over rivals (Unterweger et al., 2014). T6SS armament can be increased by acquisition of individual effector modules, or small operons encoding *vgrG/hcp/paar* and cognate effector modules or in the case of Bacteroidetes via horizontally acquired entire T6SS operons on integrative conjugative elements with near 100% identity amongst *Bacteroides* species (Allsopp et al., 2017; Coyne, Zitomersky, McGuire, Earl, & Comstock, 2014; Unterweger et al., 2014). Maintaining ancestral immunity genes or acquiring immunity but not effector genes is a good strategy to cope with T6SS‐active strains in a population at lower cost, such as the C‐type immunity gene in the aux‐1 cluster of *V. cholerae* or the recently described acquired interbacterial defence gene clusters, which encode arrays of immunity genes (Alteri et al., 2017; Kirchberger et al., 2017; Ross et al., 2019; Wexler et al., 2016).

The advantage of the T6SS in polymicrobial populations extends beyond the simple killing of competitors, preventing their use of nutrients (Figure 2). T6SS‐mediated killing that results in lysis of prey cells leads to the release of nutrients and cellular content. Studies in *V. cholerae* (Borgeaud, Metzger, Scrignari, & Blokesch, 2015) and *Acinetobacter baylyi* (Ringel, Hu, & Basler, 2017) show that regulation of the T6SS and DNA uptake is linked. This enables coordinated killing of prey and DNA acquisition from lysed bacteria, which fosters horizontal gene transfer. Incorporation of this genetic material enables the acquisition of new antimicrobial resistance genes, pathogenicity islands, or T6SS effectors/immunity pairs, all of which could provide an evolutionary advantage (Blokesch, 2017; Borgeaud et al., 2015; Kirchberger et al., 2017; Thomas, Watve, Ratcliff, & Hammer, 2017).

Acquisition of new effectors may allow successful lineages of bacteria to dominate particular environments. One example of this is within the bee, where the microbiota are dominated by nine bacterial species (including *Gilliamella apicola* and *Snodgrassella alvi*, which are T6SS^+^); however, multiple strains coexist within individual bees, hives, host species, and geographic locations. These two species contain a massive diversity of putative effectors with 1,112 identified bioinformatically within 77 strains (Steele, Kwong, Whiteley, & Moran, 2017). A subset of these effector genes was found to be upregulated in two *S. alvi* strains when grown within the bee gut, suggesting a role of the T6SS in either intrastrain competition (between *S. alvi*) or interspecies competition with other microbiota (Steele et al., 2017). This maintenance of the T6SS and diverse effectors suggests that T6SS provides an advantage and that coevolution amongst bacterial species is occurring in the bee gut. A second example is in the Hawaiian bobtail squid where intraspecies competition by *Vibrio fischeri* isolates facilitates colonisation of the light‐organ crypts. Here, T6SS^+^ strains outcompete T6SS^−^ strains within a single crypt. Contrastingly, two T6SS^+^ strains formed spatially segregated but interspersed microcolonies with a crypt. Incompatible effector modules and bacterial killing will prevent mixing similar to the Dienes lines observed in vitro for *P. mirabilis* and will highlight that T6SS fighting is occurring and shaping the population within a host (Speare et al., 2018). The T6SS can also function as a policing mechanism to enforce cooperation. In *Burkholderia thailandensis*, T6SS‐1 and a range of public goods that benefit the overall population are controlled through quorum sensing (QS). However, QS mutants have a growth advantage over wild type, as they do not produce public goods (e.g., extracellular proteases) but gain the benefits of them acting as cheaters in the population. This growth advantage can be observed in liquid coculture where the cheater population rises from 1% of the population to 50% over 3 days. However, the same population grown on solid surfaces stayed at 1% through the action of the T6SS (Majerczyk, Schneider, & Greenberg, 2016). By linking expression of public goods with antibacterial T6SS effectors, *B. thailandensis* has evolved as a hardwired regulatory system to control cheaters under defined environmental conditions.

## 4 T6SS IN PLANT‐ASSOCIATED BACTERIA

It is increasingly becoming apparent that the T6SS provides an advantage in several model systems particularly in an agricultural context. The majority of T6SSs in plant‐associated bacteria described are involved in competition (Bayer‐Santos et al., 2018; Bernal, Allsopp, Filloux, & Llamas, 2017; Decoin et al., 2014; Haapalainen et al., 2012; Ma et al., 2014) and only a few in host manipulation (Shyntum et al., 2015; L. Zhang et al., 2014; Figure 2). A recent study demonstrated that the T6SSs from the environmental bacterium *Pseudomonas putida* act as a mechanism of biocontrol eliciting antibacterial activity against a panel of resilient phytopathogens including *Xanthomonas campestris* and reduce plant symptoms during coinfection in *Nicotiana benthamiana* plants (Bernal et al., 2017). In agreement with these results, it was also shown that *Pseudomonas fluorescens* can protect potato tubers from the attack of *Pectobacterium atrosepticum* in a T6SS‐dependent manner (Decoin et al., 2014). Deletion of the T6SS from *Kosakonia* endophytes has also been demonstrated to significantly decrease plant root rhizosphere and endosphere colonisation (Mosquito et al., 2019).The environmental setting may also modulate the effectiveness of the T6SS. For example, in vitro, *Agrobacterium tumefaciens* is outcompeted by the T6SS of *P. aeruginosa*. However, in a plant coinfection assay, the T6SS of *A tumefaciens* prevails over *P. aeruginosa* to gain a competitive advantage (Ma et al., 2014). Plants species also modulate their environment to promote beneficial microbes in the root microbiome to protect against soil pathogens (Lebeis et al., 2015), and future experiments will define the contribution of the T6SS in this niche.

## 5 T6SS IN RESIDENT GUT MICROBIOTA

Given the role of the T6SS, it is reasonable to consider that this system could be capable of deeply transforming the gut microbiota, which may have a great impact on human health. The mammalian gastrointestinal tract harbours the densest microbial community currently known with dysbiosis associated with obesity, inflammatory bowel disease, and cancer. This community is numerically dominated by Gram‐positive *Firmicutes* and Gram‐negative Bacteroidales and is an attractive niche to prospect for T6SS interactions. One of the first studies to discover T6SS clusters in human gut microbiota strains was by Coyne et al. (2014). They identified the majority of the T6SS structural genes and a putative effector encoding a recombination hot spot (Rhs) protein with a deaminase domain and two putative immunities. Other studies have corroborated this with the identification of putative T6SS effector modules (e.g., zinc metalloproteases or peptidoglycan endopeptidase motifs) encoded in the vicinity of *vgrG* and *hcp* genes (Russell, Peterson, et al., 2014; Russell, Wexler, et al., 2014; D. Zhang, de Souza, Anantharaman, Iyer, & Aravind, 2012). Genomic analysis identified nine out of the 13 core Proteobacterial T6SS components (TssB/C/E/F/G/K, ClpV, VgrG and Hcp) encoded within Bacteroidetes gene clusters (Russell, Wexler, et al., 2014). Noticeably missing were the genes encoding the membrane complex proteins, but four additional genes, *tssN*, *tssO*, *tssP*, and *tssQ*, each containing at least one predicted transmembrane region, may substitute this function (Coyne, Roelofs, & Comstock, 2016). Sequence divergence of TssC resulted in the *Bacteroides* cluster to be designated the T6SS^iii^, separate from the Proteobacterial T6SS^i^ (e.g., *Pseudomonas* or *Vibrio* systems) and T6SS^ii^ (*Francisella*) clades (Russell, Wexler, et al., 2014). An analysis of the core T6SS^iii^ clusters of 205 human gut Bacteroidales revealed three distinct genetic architectures (GA1, GA2, and GA3) distinguished by conserved orientation and organisation within T6SS clusters (Coyne et al., 2016). The role of GA1 and GA2 is unknown, but putative effector–immunity pairs (e.g., several evolved PAAR proteins with putative nuclease domains) hint at it being involved in interbacterial competition (Coyne et al., 2016). Supporting this notion, multiple species encode the same effector genes within a single microbiome (Verster et al., 2017). GA1 and GA2 are shared amongst *Bacteroides* (Coyne et al., 2016; Coyne et al., 2014), whereas GA3 is exclusively found in some *Bacteroides fragilis* strains (Coyne et al., 2014). Metagenomic and strain analyses have shown a monophyletic group of *B. fragilis* within individuals, suggesting single colonisation or out‐competition events dependent on the effector–immunity pair genotypes (Verster et al., 2017). An analysis of infant microbiomes containing *B. fragilis* is significantly more likely to harbour GA3 structural genes (92%) compared with those of adults (74%), suggesting that GA3 mediates strain level competition early on in gut microbiota development (Verster et al., 2017). The presence of GA3 in *B. fragilis* is also associated with higher relative abundance of *B. fragilis* and negatively correlated with Gram‐positive Firmicutes genera (*Faecalibacterium*, *Oscillospira*, and *Ruminococcus*). As Gram‐positive bacteria have not been shown to be targets of the T6SS, the decreased abundance of these genera in GA3‐containing microbiomes is most likely due to a fitness advantage conferred to GA3^+^ *B. fragilis* and not direct action against Firmicutes (Verster et al., 2017). Several studies have also demonstrated a role of the GA3 T6SS^iii^ in the gut microbiota of mice. In competitive colonisation experiments of germ free mice, a human strain of *B. fragilis* was able to outcompete in a T6SS^iii^‐dependent manner, the gut commensal *Bacteroides thetaiotaomicron*, which lacks the T6SS (Figure 1; Russell, Wexler, et al., 2014). Chatzidaki‐Livanis et al. (2016) confirmed the expression of the system and a role in intrastrain killing as the parent outcompeted an effector–immunity pair mutant. Furthermore, a strain of *B. fragilis* can restrict colonisation of the murine host by two pathogenic strains of enterotoxigenic *Bacteroides fragilis* and reduced pathogen damage, which is associated with inflammatory bowel disease, sepsis, and colon cancer (Figure 1; Hecht et al., 2016). This T6SS antagonism between a gut resident and incoming pathogens likely contributes to gut homeostasis.

## 6 THE T6SS ARMS RACE IN THE CONTROL OF GUT MICROBIOTA/PATHOGEN BALANCE

If commensals use the T6SS to restrict invasion by intruders, it seems obvious that pathogens deploy their T6SS to invade the resident microbiota facilitating colonisation (Figure 1). *Salmonella enterica* Typhimurium is a major causative agent of human gastroenteritis. The Salmonella Pathogenicity Island Six encodes a T6SS cluster that contributes to the colonisation of mice and chickens. Recent work confirmed this role in mice and demonstrated that *S*. Typhimurium exhibits T6SS antibacterial killing of *Klebsiella oxytoca* and *Klebsiella variicola* (Sana et al., 2016). However, no killing of other Gram‐negative bacteria was observed (e.g., *Enterobacter cloacae*, *Escherichia coli*, or commensals such as *Prevotella copri*, *Parabacteroides distasonis*, and *Bifidobacterium longum*; Sana et al., 2016). This suggests the T6SS has a defined role in colonisation, which is dependent upon antibacterial activity, but *S*. Typhimurium only kills specific members of the microbiota to invade the gut. The T6SS of pathogenic *Shigella sonnei* is also required to colonise the mouse gut (Anderson, Vonaesch, Saffarian, Marteyn, & Sansonetti, 2017). This T6SS mediates out‐competition of commensal *E. coli* or *Shigella flexneri* both in vitro and in mice (Anderson et al., 2017). This confirms a role of the T6SS in host colonisation where, via direct killing, the colonisation resistance of the microbiota is overcome (Figure 1). This work also shows that *S. sonnei* has a T6SS‐mediated competitive advantage over *S. flexneri* (which lacks the T6SS) and may be contributing to its increasing global dominance over *S. flexneri* as the leading cause of Shigellosis (Anderson et al., 2017). Thus, the T6SS is an antibacterial weapon used by pathogens as a virulence factor.

Although pathogens can use the T6SS to promote colonisation, this may not be solely via killing of the microbiota and niche takeover but may also combine the host response. *Drosophila* infected with T6SS^+^ *V. cholerae* have a reduced lifespan, enhanced intestinal damage, and higher diarrheal symptoms than has T6SS^−^ infection (Fast, Kostiuk, Foley, & Pukatzki, 2018). However, removal of commensal bacteria attenuated disease, as did deletion of the fly IMD antibacterial pathway, highlighting that T6SS killing leads to secondary events that enhance pathogenicity (Fast et al., 2018). In the mammalian gut, several studies have suggested a role for the T6SS in facilitating colonisation by influencing the host response (Figures 1 and 2). Indeed, *V. cholerae* T6SS‐mediated antagonism and killing of mouse commensals was shown to drive a more acute host innate immune response with higher induction of pro‐inflammatory factors (Zhao, Caro, Robins, & Mekalanos, 2018). RNA‐seq analysis corroborates this with an upregulation of 14 host immune genes including multiple targets in the NF‐κB pathway. The authors suggest that killing of the intestinal microbiota releases microbe‐associated molecular patterns driving this response. This study also reported higher host diarrheal symptoms in mice challenged with wild type compared with the T6SS^−^. Thus, in this case, *V. cholerae* is using its antibacterial capacity to clear its niche, and this killing in turn induces a host response potentially enhancing pathogenicity and transmission (Zhao et al., 2018). In contrast, a study using the zebrafish (*Danio rerio*) model demonstrated that *V. cholerae* could manipulate the host leading to ejection of its resident microbiota (Figure 2). Delivery of the actin cross‐linking domain of *vgrG‐1* promoted host intestinal contractions, dispelling the microbiota leading to enhanced *V. cholerae* colonisation (Logan et al., 2018). Overall, these studies show that the T6SS can modulate the host gut environment for the pathogen's benefit (Figure 2).

## 7 FUTURE PERSPECTIVES AND CONCLUSIONS

For many decades, the microbial composition of the mammalian gut has been considered to play a critical role in human health, mediating everything from immune system education, regulating endocrine function, vitamin synthesis, to protecting from pathogens (Lynch & Pedersen, 2016). The examples above show that the T6SS has a role in both pathogen defence and pathogen invasion. It is attractive to consider that specific cocktails of symbiotic bacteria or even engineered T6SS^+^ commensal strains may be used as the prebiotics or probiotics in the mammalian gut, or as biocontrol agents in plants in the future. However, careful consideration is needed, as these strains may kill the microbiota as much as the pathogen. Realistically, T6SS biocontrol is something that is obviously best ready for plant pathogen control, and promising work in this area has been performed (Bernal et al., 2017; Decoin et al., 2014; Figure 2).

One additional consideration is that the control of the T6SS in many bacteria and within the intestinal conditions is likely highly complex. Experimental studies have shown that the activity of the T6SS can be influenced by host or dietary components. Bile salts, for instance, amplify *S*. Typhimurium T6SS‐mediated bacterial killing (Sana et al., 2016). Mucins or chitin have been shown to promote T6SS expression and activity in *V. cholerae* (Bachmann et al., 2015; Borgeaud et al., 2015). Intriguingly, the microbiota modify bile acids to inhibit the T6SS of pandemic *V. cholerae* (Bachmann 2015). This suggests that bacterial pathogenicity could be controlled by the addition of certain dietary components to modulate the behaviour of the microbiota.

In summary, the T6SS is present in many Gram‐negative bacteria and found in both pathogens and nonpathogens. It can perform a wide variety of functions, and mounting evidence shows a clear role in modulating the microbiota (Figures 1 and 2). This can be achieved through the direct injection of effector proteins into competing bacteria, which results in their growth inhibition or death. This death may in turn have consequences for the surrounding bacteria and the host. T6SS effectors can also directly subvert the host for their gain, and recent examples show that this can trigger changes to the microbiota. As bacteria in the human gut achieve the greatest densities recorded for any ecosystem, with an estimated 100 trillion cells, many more examples of T6SS‐associated activities are likely to be identified. Recent discoveries of the T6SS in killing fungus, that is, *Serratia marcescens* T6SS effectors Tfe1 and Tfe2 killing *Candida albicans*, *Candida glabrata*, and *Saccharomyces cerevisiae* (Trunk et al., 2018), or altering the behaviour of Gram‐positive bacteria, that is, *Pseudomonas chlororaphis* influencing sporulation of *Bacillus subtilis* (Molina‐Santiago et al., 2019), are opening unprecedented scope to this remarkable molecular weapon (Figure 2). However, the T6SS is but one of the specialised secretion systems deployed by bacteria for combat. For instance, diffusible colicins, the type IV secretion system, the type V secretion system/contact‐dependent inhibition systems, and diverse type VII secretion systems have all been shown to mediate bacterial killing in confined areas (Aoki et al., 2010; Cao, Casabona, Kneuper, Chalmers, & Palmer, 2016; Gonzalez, Sabnis, Foster, & Mavridou, 2018; Souza et al., 2015). More research will undoubtedly discover additional effectors and systems that function as bacterial weapons for the unabating microbial conflict, which results in innumerable bacterial causalities of war.

## CONFLICT OF INTERESTS

The authors have no conflicts to declare.

## References

[cmi13153-bib-0001] Ahmad, S. , Wang, B. , Walker, M. D. , Tran, H. R. , Stogios, P. J. , Savchenko, A. , … Whitney, J. C. (2019). An interbacterial toxin inhibits target cell growth by synthesizing (p)ppApp. Nature, 575, 674–678. 10.1038/s41586-019-1735-9 31695193 PMC6883173

[cmi13153-bib-0002] Allsopp, L. P. , Wood, T. E. , Howard, S. A. , Maggiorelli, F. , Nolan, L. M. , Wettstadt, S. , & Filloux, A. (2017). RsmA and AmrZ orchestrate the assembly of all three type VI secretion systems in *Pseudomonas aeruginosa* . Proceedings of the National Academy of Sciences of the United States of America, 114(29), 7707–7712. 10.1073/pnas.1700286114 28673999 PMC5530658

[cmi13153-bib-0003] Alteri, C. J. , Himpsl, S. D. , Zhu, K. , Hershey, H. L. , Musili, N. , Miller, J. E. , & Mobley, H. L. T. (2017). Subtle variation within conserved effector operon gene products contributes to T6SS‐mediated killing and immunity. PLoS Pathogens, 13(11), e1006729 10.1371/journal.ppat.1006729 29155899 PMC5714391

[cmi13153-bib-0004] Anderson, M. C. , Vonaesch, P. , Saffarian, A. , Marteyn, B. S. , & Sansonetti, P. J. (2017). *Shigella sonnei* encodes a functional T6SS used for interbacterial competition and niche occupancy. Cell Host & Microbe, 21(6), 769–776, e763. 10.1016/j.chom.2017.05.004 28618272

[cmi13153-bib-0005] Aoki, S. K. , Diner, E. J. , de Roodenbeke, C. T. , Burgess, B. R. , Poole, S. J. , Braaten, B. A. , … Low, D. A. (2010). A widespread family of polymorphic contact‐dependent toxin delivery systems in bacteria. Nature, 468(7322), 439–442. 10.1038/nature09490 21085179 PMC3058911

[cmi13153-bib-1006] Bachmann, V. , Kostiuk, B. , Unterweger, D. , Diaz‐Satizabal, L. , Ogg, S. , & Pukatzki, S. (2015). Bile salts modulate the mucin‐activated type VI secretion system of pandemic vibrio cholerae. PLOS Neglected Tropical Diseases, 9(8), e0004031 10.1371/journal.pntd.0004031 26317760 PMC4552747

[cmi13153-bib-0006] Bayer‐Santos, E. , Lima, L. d. P. , Ceseti, L. d. M. , Ratagami, C. Y. , de Santana, E. S. , da Silva, A. M. , … Alvarez‐Martinez, C. E. (2018). *Xanthomonas citri* T6SS mediates resistance to *Dictyostelium* predation and is regulated by an ECF σ factor and cognate Ser/Thr kinase. Environmental Microbiology, 20(4), 1562–1575. 10.1111/1462-2920.14085 29488354

[cmi13153-bib-0007] Bernal, P. , Allsopp, L. P. , Filloux, A. , & Llamas, M. A. (2017). The *Pseudomonas putida* T6SS is a plant warden against phytopathogens. The ISME Journal, 11(4), 972–987. 10.1038/ismej.2016.169 28045455 PMC5363822

[cmi13153-bib-0008] Bingle, L. E. , Bailey, C. M. , & Pallen, M. J. (2008). Type VI secretion: A beginner's guide. Current Opinion in Microbiology, 11(1), 3–8. 10.1016/j.mib.2008.01.006 18289922

[cmi13153-bib-0009] Blokesch, M. (2017). In and out—Contribution of natural transformation to the shuffling of large genomic regions. Current Opinion in Microbiology, 38, 22–29. 10.1016/j.mib.2017.04.001 28458094

[cmi13153-bib-0010] Borgeaud, S. , Metzger, L. C. , Scrignari, T. , & Blokesch, M. (2015). The type VI secretion system of *Vibrio cholerae* fosters horizontal gene transfer. Science, 347(6217), 63–67. 10.1126/science.1260064 25554784

[cmi13153-bib-0011] Cao, Z. , Casabona, M. G. , Kneuper, H. , Chalmers, J. D. , & Palmer, T. (2016). The type VII secretion system of *Staphylococcus aureus* secretes a nuclease toxin that targets competitor bacteria. Nature Microbiology, 2, 16183 10.1038/nmicrobiol.2016.183 PMC5325307 27723728

[cmi13153-bib-0012] Chatzidaki‐Livanis, M. , Geva‐Zatorsky, N. , & Comstock, L. E. (2016). *Bacteroides fragilis* type VI secretion systems use novel effector and immunity proteins to antagonize human gut Bacteroidales species. Proceedings of the National Academy of Sciences of the United States of America, 113(13), 3627–3632. 10.1073/pnas.1522510113 26951680 PMC4822612

[cmi13153-bib-0013] Coulthurst, S. (2019). The type VI secretion system: A versatile bacterial weapon. Microbiology, 165(5), 503–515. 10.1099/mic.0.000789 30893029

[cmi13153-bib-0014] Coyne, M. J. , Roelofs, K. G. , & Comstock, L. E. (2016). Type VI secretion systems of human gut Bacteroidales segregate into three genetic architectures, two of which are contained on mobile genetic elements. BMC Genomics, 17, 58 10.1186/s12864-016-2377-z 26768901 PMC4714493

[cmi13153-bib-0015] Coyne, M. J. , Zitomersky, N. L. , McGuire, A. M. , Earl, A. M. , & Comstock, L. E. (2014). Evidence of extensive DNA transfer between Bacteroidales species within the human gut. MBio, 5(3), e01305–e01314. 10.1128/mBio.01305-14 24939888 PMC4073490

[cmi13153-bib-0016] Decoin, V. , Barbey, C. , Bergeau, D. , Latour, X. , Feuilloley, M. G. , Orange, N. , & Merieau, A. (2014). A type VI secretion system is involved in *Pseudomonas fluorescens* bacterial competition. PLoS One, 9(2), e89411 10.1371/journal.pone.0089411 24551247 PMC3925238

[cmi13153-bib-0017] Fast, D. , Kostiuk, B. , Foley, E. , & Pukatzki, S. (2018). Commensal pathogen competition impacts host viability. Proceedings of the National Academy of Sciences of the United States of America, 115(27), 7099–7104. 10.1073/pnas.1802165115 29915049 PMC6142279

[cmi13153-bib-0018] Gonzalez, D. , Sabnis, A. , Foster, K. R. , & Mavridou, D. A. I. (2018). Costs and benefits of provocation in bacterial warfare. Proceedings of the National Academy of Sciences of the United States of America, 115(29), 7593–7598. 10.1073/pnas.1801028115 29967163 PMC6055196

[cmi13153-bib-0019] Haapalainen, M. , Mosorin, H. , Dorati, F. , Wu, R. F. , Roine, E. , Taira, S. , … Lin, N. C. (2012). Hcp2, a secreted protein of the phytopathogen *Pseudomonas syringae* pv. tomato DC3000, is required for fitness for competition against bacteria and yeasts. Journal of Bacteriology, 194(18), 4810–4822. 10.1128/JB.00611-12 22753062 PMC3430304

[cmi13153-bib-0020] Hachani, A. , Allsopp, L. P. , Oduko, Y. , & Filloux, A. (2014). The VgrG proteins are “a la carte” delivery systems for bacterial type VI effectors. The Journal of Biological Chemistry, 289(25), 17872–17884. 10.1074/jbc.M114.563429 24794869 PMC4067218

[cmi13153-bib-0021] Hachani, A. , Wood, T. E. , & Filloux, A. (2016). Type VI secretion and anti‐host effectors. Current Opinion in Microbiology, 29, 81–93. 10.1016/j.mib.2015.11.006 26722980

[cmi13153-bib-0022] Hecht, A. L. , Casterline, B. W. , Earley, Z. M. , Goo, Y. A. , Goodlett, D. R. , & Bubeck Wardenburg, J. (2016). Strain competition restricts colonization of an enteric pathogen and prevents colitis. EMBO Reports, 17(9), 1281–1291. 10.15252/embr.201642282 27432285 PMC5007561

[cmi13153-bib-0023] Hood, R. D. , Singh, P. , Hsu, F. , Guvener, T. , Carl, M. A. , Trinidad, R. R. , … Mougous, J. D. (2010). A type VI secretion system of *Pseudomonas aeruginosa* targets a toxin to bacteria. Cell Host & Microbe, 7(1), 25–37. 10.1016/j.chom.2009.12.007 20114026 PMC2831478

[cmi13153-bib-0024] Jana, B. , Fridman, C. M. , Bosis, E. , & Salomon, D. (2019). A modular effector with a DNase domain and a marker for T6SS substrates. Nature Communications, 10(1), 3595 10.1038/s41467-019-11546-6 PMC6688995 31399579

[cmi13153-bib-0025] Jiang, F. , Waterfield, N. R. , Yang, J. , Yang, G. , & Jin, Q. (2014). A *Pseudomonas aeruginosa* type VI secretion phospholipase D effector targets both prokaryotic and eukaryotic cells. Cell Host & Microbe, 15(5), 600–610. 10.1016/j.chom.2014.04.010 24832454

[cmi13153-bib-0026] Kirchberger, P. C. , Unterweger, D. , Provenzano, D. , Pukatzki, S. , & Boucher, Y. (2017). Sequential displacement of type VI secretion system effector genes leads to evolution of diverse immunity gene arrays in *Vibrio cholerae* . Scientific Reports, 7, 45133 10.1038/srep45133 28327641 PMC5361080

[cmi13153-bib-0027] LaCourse, K. D. , Peterson, S. B. , Kulasekara, H. D. , Radey, M. C. , Kim, J. , & Mougous, J. D. (2018). Conditional toxicity and synergy drive diversity among antibacterial effectors. Nature Microbiology, 3(4), 440–446. 10.1038/s41564-018-0113-y PMC5876133 29459733

[cmi13153-bib-0028] Lebeis, S. L. , Paredes, S. H. , Lundberg, D. S. , Breakfield, N. , Gehring, J. , McDonald, M. , … Dangl, J. L. (2015). Plant microbiome. Salicylic acid modulates colonization of the root microbiome by specific bacterial taxa. Science, 349(6250), 860–864. 10.1126/science.aaa8764 26184915

[cmi13153-bib-0029] Lin, J. , Zhang, W. , Cheng, J. , Yang, X. , Zhu, K. , Wang, Y. , … Shen, X. (2017). A *Pseudomonas* T6SS effector recruits PQS‐containing outer membrane vesicles for iron acquisition. Nature Communications, 8, 14888 10.1038/ncomms14888 PMC5379069 28348410

[cmi13153-bib-0030] Logan, S. L. , Thomas, J. , Yan, J. , Baker, R. P. , Shields, D. S. , Xavier, J. B. , … Parthasarathy, R. (2018). The *Vibrio cholerae* type VI secretion system can modulate host intestinal mechanics to displace gut bacterial symbionts. Proceedings of the National Academy of Sciences of the United States of America, 115(16), E3779–E3787. 10.1073/pnas.1720133115 29610339 PMC5910850

[cmi13153-bib-0031] Lynch, S. V. , & Pedersen, O. (2016). The human intestinal microbiome in health and disease. The New England Journal of Medicine, 375(24), 2369–2379. 10.1056/NEJMra1600266 27974040

[cmi13153-bib-0032] Ma, L. S. , Hachani, A. , Lin, J. S. , Filloux, A. , & Lai, E. M. (2014). *Agrobacterium tumefaciens* deploys a superfamily of type VI secretion DNase effectors as weapons for interbacterial competition in planta. Cell Host & Microbe, 16(1), 94–104. 10.1016/j.chom.2014.06.002 24981331 PMC4096383

[cmi13153-bib-0033] Majerczyk, C. , Schneider, E. , & Greenberg, E. P. (2016). Quorum sensing control of type VI secretion factors restricts the proliferation of quorum‐sensing mutants. eLife, 5, e14712 10.7554/eLife.14712 27183270 PMC4868534

[cmi13153-bib-0034] Miyata, S. T. , Unterweger, D. , Rudko, S. P. , & Pukatzki, S. (2013). Dual expression profile of type VI secretion system immunity genes protects pandemic *Vibrio cholerae* . PLoS Pathogens, 9(12), e1003752 10.1371/journal.ppat.1003752 24348240 PMC3857813

[cmi13153-bib-0035] Molina‐Santiago, C. , Pearson, J. R. , Navarro, Y. , Berlanga‐Clavero, M. V. , Caraballo‐Rodriguez, A. M. , Petras, D. , … Romero, D. (2019). The extracellular matrix protects Bacillus subtilis colonies from *Pseudomonas* invasion and modulates plant co‐colonization. Nature Communications, 10(1), 1919 10.1038/s41467-019-09944-x PMC6478825 31015472

[cmi13153-bib-0036] Mosquito, S. , Bertani, I. , Licastro, D. , Compant, S. , Myers, M. P. , Hinarejos, E. , … Venturi, V. (2019). In planta colonization and role of T6SS in two rice Kosakonia endophytes. Molecular Plant‐Microbe Interactions. 10.1094/mpmi-09-19-0256-r 31609645

[cmi13153-bib-0037] Nguyen, V. S. , Douzi, B. , Durand, E. , Roussel, A. , Cascales, E. , & Cambillau, C. (2018). Towards a complete structural deciphering of type VI secretion system. Current Opinion in Structural Biology, 49, 77–84. 10.1016/j.sbi.2018.01.007 29414515

[cmi13153-bib-0038] Nolan, L. M. , Cain, A. K. , Manoli, E. , Sainz‐Polo, M. A. , Dougan, G. , Mavridou, D. A. I. , … Filloux, A. (2019). Discovery of a *Pseudomonas aeruginosa* type VI secretion 1 system toxin targeting bacterial protein synthesis using a global genomics approach. bioRxiv. 10.1101/733030

[cmi13153-bib-0039] Pissaridou, P. , Allsopp, L. P. , Wettstadt, S. , Howard, S. A. , Mavridou, D. A. I. , & Filloux, A. (2018). The *Pseudomonas aeruginosa* T6SS‐VgrG1b spike is topped by a PAAR protein eliciting DNA damage to bacterial competitors. Proceedings of the National Academy of Sciences of the United States of America, 115(49), 12519–12524. 10.1073/pnas.1814181115 30455305 PMC6298103

[cmi13153-bib-0040] Ringel, P. D. , Hu, D. , & Basler, M. (2017). The role of type VI secretion system effectors in target cell lysis and subsequent horizontal gene transfer. Cell Reports, 21(13), 3927–3940. 10.1016/j.celrep.2017.12.020 29281838

[cmi13153-bib-0041] Ross, B. D. , Verster, A. J. , Radey, M. C. , Schmidtke, D. T. , Pope, C. E. , Hoffman, L. R. , … Mougous, J. D. (2019). Human gut bacteria contain acquired interbacterial defence systems. Nature, 575, 224–228. 10.1038/s41586-019-1708-z 31666699 PMC6938237

[cmi13153-bib-0042] Russell, A. B. , Hood, R. D. , Bui, N. K. , LeRoux, M. , Vollmer, W. , & Mougous, J. D. (2011). Type VI secretion delivers bacteriolytic effectors to target cells. Nature, 475(7356), 343–347. 10.1038/nature10244 21776080 PMC3146020

[cmi13153-bib-0043] Russell, A. B. , LeRoux, M. , Hathazi, K. , Agnello, D. M. , Ishikawa, T. , Wiggins, P. A. , … Mougous, J. D. (2013). Diverse type VI secretion phospholipases are functionally plastic antibacterial effectors. Nature, 496(7446), 508–512. 10.1038/nature12074 23552891 PMC3652678

[cmi13153-bib-0044] Russell, A. B. , Peterson, S. B. , & Mougous, J. D. (2014). Type VI secretion system effectors: Poisons with a purpose. Nature Reviews. Microbiology, 12(2), 137–148. 10.1038/nrmicro3185 24384601 PMC4256078

[cmi13153-bib-0045] Russell, A. B. , Singh, P. , Brittnacher, M. , Bui, N. K. , Hood, R. D. , Carl, M. A. , … Mougous, J. D. (2012). A widespread bacterial type VI secretion effector superfamily identified using a heuristic approach. Cell Host & Microbe, 11(5), 538–549. 10.1016/j.chom.2012.04.007 22607806 PMC3358704

[cmi13153-bib-0046] Russell, A. B. , Wexler, A. G. , Harding, B. N. , Whitney, J. C. , Bohn, A. J. , Goo, Y. A. , … Mougous, J. D. (2014). A type VI secretion‐related pathway in Bacteroidetes mediates interbacterial antagonism. Cell Host & Microbe, 16(2), 227–236. 10.1016/j.chom.2014.07.007 25070807 PMC4136423

[cmi13153-bib-0047] Sana, T. G. , Flaugnatti, N. , Lugo, K. A. , Lam, L. H. , Jacobson, A. , Baylot, V. , … Monack, D. M. (2016). *Salmonella* Typhimurium utilizes a T6SS‐mediated antibacterial weapon to establish in the host gut. Proceedings of the National Academy of Sciences of the United States of America, 113(34), E5044–E5051. 10.1073/pnas.1608858113 27503894 PMC5003274

[cmi13153-bib-0048] Schell, M. A. , Ulrich, R. L. , Ribot, W. J. , Brueggemann, E. E. , Hines, H. B. , Chen, D. , … Deshazer, D. (2007). Type VI secretion is a major virulence determinant in *Burkholderia mallei* . Molecular Microbiology, 64(6), 1466–1485. 10.1111/j.1365-2958.2007.05734.x 17555434

[cmi13153-bib-0049] Shyntum, D. Y. , Theron, J. , Venter, S. N. , Moleleki, L. N. , Toth, I. K. , & Coutinho, T. A. (2015). *Pantoea ananatis* utilizes a type VI secretion system for pathogenesis and bacterial competition. Molecular Plant‐Microbe Interactions, 28(4), 420–431. 10.1094/MPMI-07-14-0219-R 25411959

[cmi13153-bib-0050] Souza, D. P. , Oka, G. U. , Alvarez‐Martinez, C. E. , Bisson‐Filho, A. W. , Dunger, G. , Hobeika, L. , … Farah, C. S. (2015). Bacterial killing via a type IV secretion system. Nature Communications, 6, 6453 10.1038/ncomms7453 25743609

[cmi13153-bib-0051] Speare, L. , Cecere, A. G. , Guckes, K. R. , Smith, S. , Wollenberg, M. S. , Mandel, M. J. , … Septer, A. N. (2018). Bacterial symbionts use a type VI secretion system to eliminate competitors in their natural host. Proceedings of the National Academy of Sciences of the United States of America, 115(36), E8528–E8537. 10.1073/pnas.1808302115 30127013 PMC6130350

[cmi13153-bib-0052] Steele, M. I. , Kwong, W. K. , Whiteley, M. , & Moran, N. A. (2017). Diversification of type VI secretion system toxins reveals ancient antagonism among bee gut microbes. MBio, 8(6), e01630‐17 10.1128/mBio.01630-17 29233893 PMC5727410

[cmi13153-bib-0053] Thomas, J. , Watve, S. S. , Ratcliff, W. C. , & Hammer, B. K. (2017). Horizontal gene transfer of functional type VI killing genes by natural transformation. MBio, 8(4), e00654‐17 10.1128/mBio.00654-17 28743812 PMC5527308

[cmi13153-bib-0054] Ting, S. Y. , Bosch, D. E. , Mangiameli, S. M. , Radey, M. C. , Huang, S. , Park, Y. J. , … Mougous, J. D. (2018). Bifunctional immunity proteins protect bacteria against FtsZ‐targeting ADP‐ribosylating toxins. Cell, 175(5), 1380–1392, e1314. 10.1016/j.cell.2018.09.037 30343895 PMC6239978

[cmi13153-bib-0055] Trunk, K. , Peltier, J. , Liu, Y. C. , Dill, B. D. , Walker, L. , Gow, N. A. R. , … Coulthurst, S. J. (2018). The type VI secretion system deploys antifungal effectors against microbial competitors. Nature Microbiology, 3(8), 920–931. 10.1038/s41564-018-0191-x PMC6071859 30038307

[cmi13153-bib-0056] Unterweger, D. , Miyata, S. T. , Bachmann, V. , Brooks, T. M. , Mullins, T. , Kostiuk, B. , … Pukatzki, S. (2014). The *Vibrio cholerae* type VI secretion system employs diverse effector modules for intraspecific competition. Nature Communications, 5, 3549 10.1038/ncomms4549 PMC3988814 24686479

[cmi13153-bib-0057] Verster, A. J. , Ross, B. D. , Radey, M. C. , Bao, Y. , Goodman, A. L. , Mougous, J. D. , & Borenstein, E. (2017). The landscape of type VI secretion across human gut microbiomes reveals its role in community composition. Cell Host & Microbe, 22(3), 411–419, e414 10.1016/j.chom.2017.08.010 28910638 PMC5679258

[cmi13153-bib-0058] Wexler, A. G. , Bao, Y. , Whitney, J. C. , Bobay, L. M. , Xavier, J. B. , Schofield, W. B. , … Goodman, A. L. (2016). Human symbionts inject and neutralize antibacterial toxins to persist in the gut. Proceedings of the National Academy of Sciences of the United States of America, 113(13), 3639–3644. 10.1073/pnas.1525637113 26957597 PMC4822603

[cmi13153-bib-0059] Whitney, J. C. , Beck, C. M. , Goo, Y. A. , Russell, A. B. , Harding, B. N. , De Leon, J. A. , … Mougous, J. D. (2014). Genetically distinct pathways guide effector export through the type VI secretion system. Molecular Microbiology, 92(3), 529–542. 10.1111/mmi.12571 24589350 PMC4049467

[cmi13153-bib-0060] Whitney, J. C. , Chou, S. , Russell, A. B. , Biboy, J. , Gardiner, T. E. , Ferrin, M. A. , … Mougous, J. D. (2013). Identification, structure, and function of a novel type VI secretion peptidoglycan glycoside hydrolase effector–immunity pair. The Journal of Biological Chemistry, 288(37), 26616–26624. 10.1074/jbc.M113.488320 23878199 PMC3772208

[cmi13153-bib-0061] Whitney, J. C. , Quentin, D. , Sawai, S. , LeRoux, M. , Harding, B. N. , Ledvina, H. E. , … Mougous, J. D. (2015). An interbacterial NAD(P)(+) glycohydrolase toxin requires elongation factor Tu for delivery to target cells. Cell, 163(3), 607–619. 10.1016/j.cell.2015.09.027 26456113 PMC4624332

[cmi13153-bib-0062] Wood, T. E. , Howard, S. A. , Forster, A. , Nolan, L. M. , Manoli, E. , Bullen, N. P. , … Filloux, A. (2019). The *Pseudomonas aeruginosa* T6SS delivers a periplasmic toxin that disrupts bacterial cell morphology. Cell Reports, 29(1), 187–201, e187. 10.1016/j.celrep.2019.08.094 31577948 PMC6899460

[cmi13153-bib-0063] Zhang, D. , de Souza, R. F. , Anantharaman, V. , Iyer, L. M. , & Aravind, L. (2012). Polymorphic toxin systems: Comprehensive characterization of trafficking modes, processing, mechanisms of action, immunity and ecology using comparative genomics. Biology Direct, 7, 18 10.1186/1745-6150-7-18 22731697 PMC3482391

[cmi13153-bib-0064] Zhang, L. , Xu, J. , Xu, J. , Zhang, H. , He, L. , & Feng, J. (2014). TssB is essential for virulence and required for type VI secretion system in *Ralstonia solanacearum* . Microbial Pathogenesis, 74, 1–7. 10.1016/j.micpath.2014.06.006 24972114

[cmi13153-bib-0065] Zhao, W. , Caro, F. , Robins, W. , & Mekalanos, J. J. (2018). Antagonism toward the intestinal microbiota and its effect on *Vibrio cholerae* virulence. Science, 359(6372), 210–213. 10.1126/science.aap8775 29326272 PMC8010019

